# Mechanical Devices for Cleft Palate

**Published:** 1902-11-15

**Authors:** H. J. Jaulusz


### MECHANICAL DEVICES FOR CLEFT PALATE.

BY DR. H. J. JAULUSZ.

Read at a Meeting of the Southwestern Michigan Dental Society, Sept. 10, 1902.

I am very grateful for the opportunity accorded me through the courtesy of your secretary, who extended the invitation to me to be represented to day and to have the privilege of addressing you.

The subject I wish to speak about is, one which permits of a comprehensive description; it being an innovation in the nature of a mechanical contrivance for certain forms of cleft palate. I will give you as clear an explanation of the contrivance as I possibly can.

This work, in my estimation, fills a long felt want in the branch of dentistry, of which I make a specialty. I am not looking for glory nor praise, but since mankind has been injected with the virus of self-conceit, a recognition, is the compensation for which I look. I should like it if you have the opportunity in your practice to employ my method for suppurative cleft palate. It is by no means new to the profession, especially to those who come in frequent contact with patients suffering with cleft palate a single illustration is sufficient. I assure you that from my experience in the introduction of this contrivance, I have secured success in abundance. Years ago, the lamented Dr. Atkinson—the crown and pride of the dental profession, whose acquaintance I made very soon after landing from a country where dentistry is still in its infancy; and at which time I began to manipulate and invent various articles for the use of the profession, said to me, "young man, you are on the proper road to make a name for yourself, if you can make your work practicable."

It is not my purpose to appear as a philantropist, nor as a good samaritan, but I do say with pride, that I have never attempted to patent any of my inventions. I owe it a duty of my brethren, to make as much of my little ability as I can. Upon the field of dental science, I do not believe that anything is of such interest as cleft palate work. To make a poor miserable sufferer more comfortable, who through his own or the sin of his forefathers becomes inoculated with a dreaded disease "syphilis."

I may pride myself, when I say, I have made many more comfortable and life also worth living. The first case I show you by a plaster model the condition of the maxilla five years ago, and another model illustrates the condition last February. Through the kindness of Dr. Ottolengui, Editor of the *Items of Interest*, the profession has been informed in the March 1902 number, page 168, of this very interesting case.

Should you have a case of this nature, proceed as follows: After examining the condition of the abnormal cavity of the palate wash the same with a full strength solution of glyco-thymoline. After this procedure take a quantity of absorbant cotton and place the same in sweet oil and wipe out the cavity, now take a sheet of wax and soften it and place the same gently into the cavity, remove again, cool and replace it filled level to surface of natural palate with wax. Take a piece of cloth and immerse it in hot water and smooth the wax to a uniform contour with the rest or healthy portion of the palate. Make several wire hooks and stick them into the wax and proceed to take the impression as usually. Remove the impression, being careful that the wax which filled the cavity be removed with the impression proper cleanse and pour the model. After you have removed the model from the impression boil out the wax and you have an exact impression and condition of your case. Dry the model after it is sufficiently cool and fill the cleft with wax and make a babbitt-metal cast, as you would for an ordinary metal plate. By the way, I wish to call your special attention to the fact, that metal plates are more desireable in this class of work than rubber. They can be kept cleaner and more wholesome. The details of the appliance for holding the sponge will need to be worked out in accordance with the demand of each individual case.

Patient No. 1 was inoculated with syphilis many years ago, but living in a country where physicians were not sufficiently acquainted with the treatment of syphilis, he neglected the treatment. He could not speak plainly, nor articulate his vocabulary properly prior to the insertion of this plate, but since wearing the plate, the disagreeable discharge of mucus ceased running into his throat. The sponge, which is attached by means of the various appliances to the upper side of the plate, absorbs all secretions. Most of these cases are about of the same nature, and in all cases you can apply the same method. The method is to attach to the upper or nasal surface of the metal plates some form of clasp, hook or screws which will retain a small sponge the size of the cavity in place, so that the sponge may be readily removed as often as may be necessary and without difficulty by the patient.

*Case II.*—A young man acquired syphilis a dozen years ago; the disease made a very rapid progress, attacking very savagely the maxilla. In addition to this trouble, an ulcer made its appearance on the uvula about a year ago. It was rather difficult to keep a dressing on the uvula owing to the constant muscular movements of the soft palate. I extended a strip of the plate back to the uvula and soldered a sponge holder to it and this appliance in addition to the cleft plate held a dressing in place, which was very successful, the ulcer healed in fine condition.

*Case III.*—A gentleman called on me—having been recommended by a well known dentist of New York—for the purpose of remedying his trouble, but as his time was very limited, I could not make him a regular appliance, but I knew the need and use of a sponge to absorb the mucus, so I soldered with soft solder to his plate one of the little contrivances which are used to hold the necktie in place. I soldered this to his metal plate as a temporary appliance and since then he has returned and I have made him a proper plate. He was so delighted with my work that he brought his daughter, a young lady about 26 years old, suffering from acquired syphilis. The young lady told me that she was never blessed with a set of teeth. Singularly, this woman is to be married very shortly. I believe a law should be enacted preventing people suffering from this disease from intermarrying. I do not wish to appear as a prophet, but I dare say, that should these things go on, a great deal of misery will be the outcome.

*Case IV.*—A young man, who, but a few years ago, was a healthy robust working man, was inoculated with syphilis. The disease has treated him step-mother-like, destroying both sides of the hard palate.

